# Health professionals’ acceptance of mobile-based clinical guideline application in a resource-limited setting: using a modified UTAUT model

**DOI:** 10.1186/s12909-024-05680-z

**Published:** 2024-06-25

**Authors:** Addisalem Workie Demsash, Mulugeta Hayelom Kalayou, Agmasie Damtew Walle

**Affiliations:** 1 Health Informatics Department, Debre Berhan University, Asrat Woldeyes Health Science Campus, P.O. Box 445, Debre Birhan, Ethiopia; 2 College of Health Science, Health Informatics Department, Wollo University, Dessie, Ethiopia

**Keywords:** Mobile device, Clinical guideline, Acceptance, Application, UTAUT model

## Abstract

**Introduction:**

Clinical guidelines are crucial for assisting health professionals to make correct clinical decisions. However, manual clinical guidelines are not accessible, and this increases the workload. So, a mobile-based clinical guideline application is needed to provide real-time information access. Hence, this study aimed to assess health professionals’ intention to accept mobile-based clinical guideline applications and verify the unified theory of acceptance and technology utilization model.

**Methods:**

Institutional-based cross-sectional study design was used among 803 study participants. The sample size was determined based on structural equation model parameter estimation criteria with stratified random sampling. Amos version 23 software was used for analysis. Internal consistency of latent variable items, and convergent and divergent validity, were evaluated using composite reliability, AVE, and a cross-loading matrix. Model fitness of the data was assessed based on a set of criteria, and it was achieved. P-value < 0.05 was considered for assessing the formulated hypothesis.

**Results:**

Effort expectancy and social influence had a significant effect on health professionals’ attitudes, with path coefficients of (**β = 0.61, P-value < 0.01**), and (**β = 0.510, P-value < 0.01**) respectively. Performance expectancy, facilitating condition, and attitude had significant effects on health professionals’ acceptance of mobile-based clinical guideline applications with path coefficients of (**β = 0.37, P-value < 0.001**), (**β = 0.44, P-value < 0.001**) and (**β = 0.57, P-value < 0.05**) respectively. Effort expectancy and social influence were mediated by attitude and had a significant partial relationship with health professionals’ acceptance of mobile-based clinical guideline application with standardized estimation coefficients of (**β = 0.22, P-value = 0.027**), and (**β = 0.19, P-value = 0.031**) respectively. All the latent variables accounted for 57% of health professionals’ attitudes, and latent variables with attitudes accounted for 63% of individuals’ acceptance of mobile-based clinical guideline applications.

**Conclusions:**

The unified theory of acceptance and use of the technology model was a good model for assessing individuals’ acceptance of mobile-based clinical guidelines applications. So, enhancing health professionals’ attitudes, and computer literacy through training are needed. Mobile application development based on user requirements is critical for technology adoption, and people’s support is also important for health professionals to accept and use the application.

**Supplementary Information:**

The online version contains supplementary material available at 10.1186/s12909-024-05680-z.

## Introduction

Clinical practice guidelines are methodically developed statements to assist health professionals and patients’ decisions about suitable healthcare for specific clinical conditions. When it comes to a particular therapy, diagnosis, and pharmaceutical processes in patient care, clinical practice guidelines play a major role [1]. The medical guideline isn’t a fixed protocol that must be followed; it is also a recommendation for healthcare professionals to consider for correct patient diagnosis and treatment [2], as well as a written document that swiftly offers technical assistance, advice on the definition and operationalization of medical terms, and certain aspects of planning for implementation and evaluation [3].

A clinical guideline has several benefits and opportunities for healthcare practitioners, institutions, and patients. It enhances health professionals’ communications and evidence-based practice [4–6]. It serves as the same standard in all health institutions for diagnosis and treatment to ensure the consistency of patient care and is critical for quality audits and evaluations [7]. Plus, clinical guidelines are part of the work of health professionals’ consultants and are fertile for the care of patients as references for health professionals to access the right information when and where needed.

Additionally, well-trained health professionals are not equally accessible in all health institutions in low-income countries; their educational and training qualifications vary; providing the training is expensive [8], their job function performance is limited, and treatment and medication errors are common in healthcare practice [9, 10]. Therefore, clinical guidelines are critical to solving such kinds of problems. However, it is manual (paper-based) and vigorously promoted as a means to improve the effectiveness of the healthcare system, patient outcomes, and healthcare costs [11]. It needs huge physical space for storage, is exposed to fire and easily lost, and is inaccessible to health professionals [12]. The manuals are poorly designed, present incomplete explanations that are difficult to read, have comprehension levels beyond the user’s capabilities, lack explicit workflow, and increase the user’s workload [13–15]. Moreover, the clinical guidelines are available in voluminous text files and are very laborious and time-consuming to access [16]. Therefore, this may promote distorted health information so that health professionals cannot access appropriate guidelines at the point of patient care [17].

Currently, technology has become commonplace in a healthcare setting, and there has been rapid growth in the development of medical application software [18–20]. Several platforms are available to assist health professionals, such as patient information management and access, communication, and consulting [21, 22], reference and information gathering, distance medical education and training, and clinical support systems for accurate decision-making [23, 24]. Mobile devices and mobile health applications are also among the fastest and most convenient ways for health professionals to access educational materials, including medication information, electronic clinical guidelines, and books [25, 26].

In Sweden, a variety of wireless technologies such as mobile computing, wireless networks, and global positioning systems have been applied to ambulance care [27], and these are also functional for emergency patient care in the Netherlands [28]. In Finland, an authorized and secured mobile healthcare services system was tested in 2003 and is available nationwide, that is used for consultation, electronic prescription, and easy access to health information via mobile devices [29]. Though information technologies are an essential tool that fosters and promotes progress in healthcare and drastically reforms healthcare practices, the healthcare system in low-income countries is recognized as having lagged behind other industries in the use and adoption of information communication technologies [30, 31]. Therefore, mobile-based clinical guidelines applications are used as job aid tools for real-time information and knowledge access and update, improving health professionals’ performance by directing and guiding in an interactive and structured manner using mobile devices [32, 33].

In low-income countries, mobile devices are not widely utilized for daily healthcare practice in terms of providing real-time access to clinical guidelines for healthcare practitioners. Mobile-based clinical guidelines add valuable functions for health professionals in terms of presenting completed information and reducing their workload. However, healthcare professionals did not adequately use mobile devices and related applications for healthcare systems. The development of mobile-based medical applications and technology-based healthcare practices is still in its premature stages [34]. Information and communication technologies (ICT) are efficient and effective in many industries. However, they are not yet fully implemented and integrated into existing patient care systems, and healthcare institutions, particularly professionals are noticeably lagging in accepting and adopting technologies [35].

The lack of acceptance due to a lack of awareness towards mobile-based clinical guideline application, a lack of system user self-efficacy, a lack of outcome expectations, health professionals’ attitudes and perceptions [36, 37], lack of commitment and motivation [34, 38], lack of organizational support, the constructs of the technology acceptance model (TAM) [34, 38], and socioeconomic characteristics of the health professionals [39] are factors for acceptance and utilization of mobile-based clinical guidelines applications in the healthcare practice. So, understanding why healthcare professionals could not accept and use mobile-based healthcare systems would accelerate hospital competition and enhance the acceptance and utilization of mobile devices and the Internet in healthcare practices [27, 40]. It is also important to provide critical insight for the development of effective strategies to increase the efficiency and effectiveness of healthcare personnel [41, 42].

In Ethiopia, several eHealth technologies that could support healthcare practices have been introduced. Electronic medical record system, district health information system version 2 (DHIS2), routine health information system [43, 44], interactive voice response system, patient appointment reminder system, electronic community-based health information system, and international classification of disease version 10 (ICD-10) for disease coding and classification are mainly introduced in Ethiopia to support the healthcare system process, enhance documentation and reporting system [45, 46]. The implementation process of the systems is extremely costly and uncertain. As a result, eHealth technology adoption and dissemination in Ethiopia are still in their infancy [39, 47, 48]. So, there is a high demand for an easily accessible electronic system for daily healthcare practice and challenges to patient care [47]. Therefore, before starting the mobile-based clinical guideline implementation process, creating a clear understanding of the gap that exists between the manual, and the benefits of mobile-based clinical guidelines would create awareness for system users. This would also provide an effective and efficient system development process that could make the practitioners agree and be willing to accept mobile-based clinical guidelines [49].

According to our literature searching skills and the information we have, there are no adequate studies about health professionals’ acceptance of mobile-based clinical guidelines in Ethiopia. Therefore, this study would have implications for policy design, facilitating dissemination updating clinical guidelines, receiving users’ feedback, and enhancing the clinical guideline standards. This study is critically significant for health professionals’ theoretical learning, enhancing understanding that mobile-based clinical guidelines application would help them access previous work experience, and patient history to provide accurate and consistent patient care practice.

Hence, health policy implementers and practitioners were informed that medical errors could be reduced, the accuracy of patient care could be ensured, and health professionals could be easily supported by the hand-held clinical guideline application. The study would serve as a framework for further similar research. Therefore, this study aimed to assess health professionals’ acceptance of mobile-based clinical guideline applications and test a unified theory of acceptance and technology utilization (UTAUT) model.

## Theoretical background and hypothesis development

**Fig. 1:**
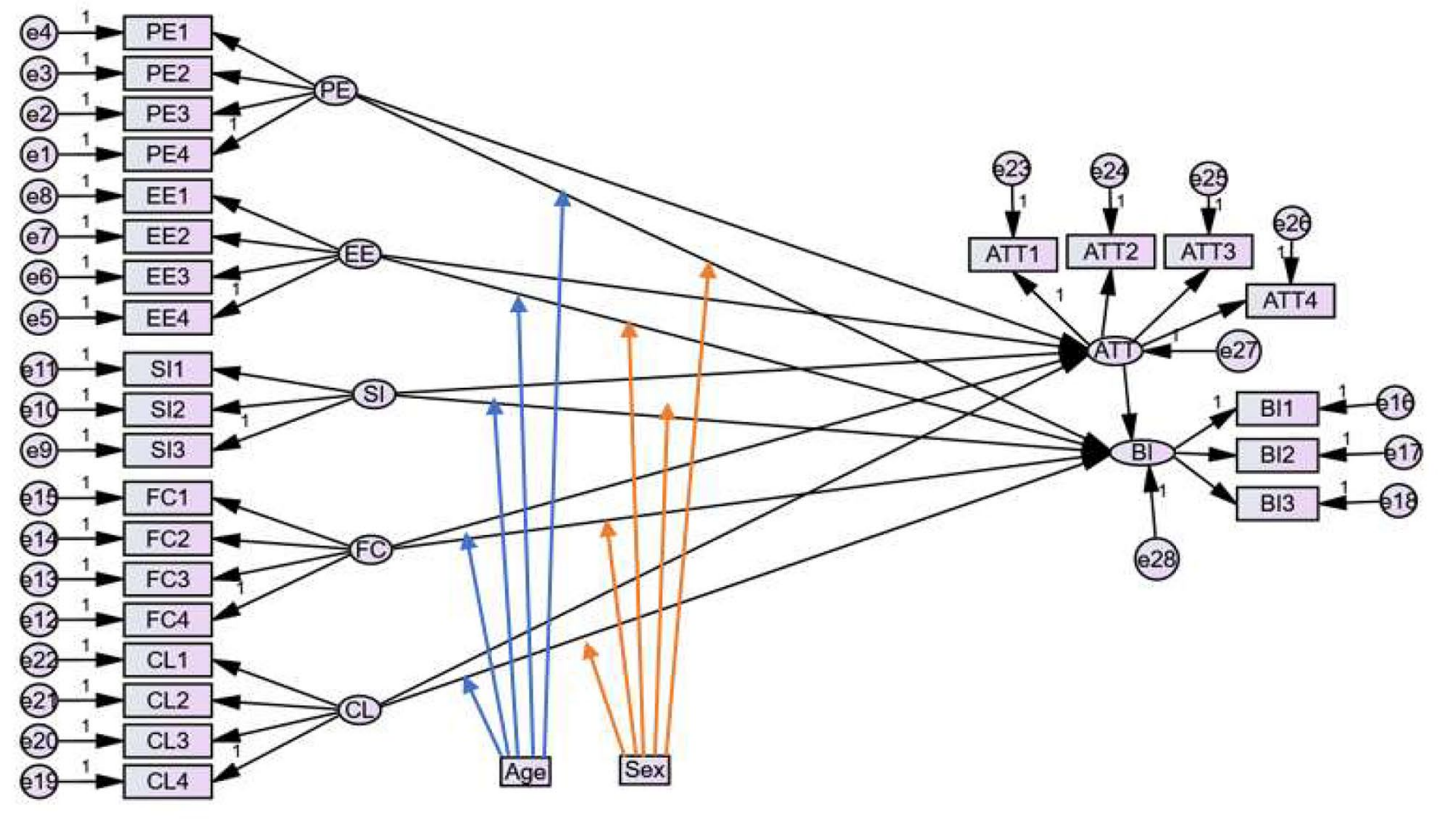
Modified theoretical acceptance and use of technology model

In the last decade, numerous theoretical models have been projected to assess and explain the end-user’s acceptance of information and communication technology (ICT) [50]. A unified theory of acceptance and use of technology (UTAUT) is one of the known theoretical models that is extensively used and practically tested on a wide range of ICT applications according to the end-users viewpoint [51]. UTAUT is a combination of activity theory and technology acceptance models (TAM) and has been constructed as a framework to study end-users acceptance and use of new ICT applications [52]. The UTAUT model proposed that the actual acceptance and use of technology are affected by end-users behavioural intentions (BI) [53]. The UTAUT model is an extension of other models and therefore has a strong ability to explain the acceptance and use of technology as compared with other single models [54, 55]. The UTAUT model consists of four key construct elements that directly affect the users’ BI of acceptance of mobile-based clinical guideline applications: performance expectancy, effort expectancy, social influence, and facilitating conditions [51, 56]. BI is additionally affected by individuals’ attitudes toward acceptance and use of new ICT applications, which are directly affected by the four key constructs [39]. Age, sex, and experience were used as moderator factors in this study. Various information communication technologies, mobile-based information systems, and integrated components that would test the health professional’s behavioural intention toward acceptance of mobile-based clinical guidelines were considered for the articulation of the study. The modified UTAUT model was applied to test the user’s acceptance, and intention to use various technologies for healthcare practice in low-income countries. For instance, a study conducted in Burundi states that the UTAUT model is critical to explaining users’ intention to adopt mobile-based information systems [57]. In Tanzania, the UTAUT model is used to test accredited drug dispensing outlet programs and to identify factors that would impact system users [58]. In Ethiopia, various studies confirmed that the modified UTAUT model is suitable for the acceptance of electronic medical and personal health record systems among the health professionals perspective [59, 60], the adoption of e-learning [61], and the sustainable adoption of the eHealth system [39]. Moderators such as age [62, 63], sex [64–66], and experience could influence the model predictors and health professionals’ intention to accept mobile-based clinical guideline applications. The practical utilization of mobile-based clinical guideline applications in resource-limited settings has not been initiated and implemented in Ethiopia. Therefore, actual system use was not measured, and the experience was removed from the structural equation model analysis as the study participants had no familiarity with mobile-based clinical guidelines application. The actual modified UTAUT model framework of the study is presented in Fig. 1.

Based on the above actual UTAUT model, the following hypotheses were developed.

### Performance expectancy

Performance expectance (**PE**) is the degree to which individuals believe that using ICT applications has the benefit of enhancing one’s job performance [67]. PE is identified as a strong determinant of BI’s use of ICT applications in different settings [67–69]. Many studies have proven that using mobile-based applications in healthcare practice has benefits for one’s health and enhances health practitioners’ job performance [70–72]. Performance expectance is one of the possible predictors for mHealth adoption in Burundi [57]. However, a study in Australia confirmed that performance expectance does not affect individuals’ intention to use cloud-based mHealth services [73]. Accordingly, the following hypothesis was developed.

#### H1

**PE** has positive effects on health professionals’ attitudes toward mobile-based clinical guideline applications.

#### H2

**PE** has a positive effect on health professionals’ BI of mobile-based clinical guideline application acceptance.

### Effort expectancy

Effort expectancy (EE) is one of the crucial elements of technology acceptance in the UTAUT model and it answers “How much the new ICT technology is easy to use?” [56]. Studies depicted that EE influences users BI to accept and use new ICT applications, and it does not require efforts to work through new technology [39, 74, 75]. A study in a low-resource setting shows that effort expectancy is a key determinant of health professionals’ intention toward telemedicine [76]. Another study in Canada shows that information systems and technology acceptance and use are significantly influenced by effort expectancy [77]. Therefore, the following hypothesis was developed.

#### H3

**EE** has significant values on health professionals’ attitudes toward mobile-based clinical guideline applications.

#### H4

**EE** has significant effects on health professionals’ BI to accept mobile-based clinical guideline applications.

### Social influence

Social influence (**SI**) is the degree to which system users assume that others would encourage them to use the new ICT technology [56]. According to studies, **SI** has a positive association with BI to accept and use new mobile health applications for healthcare practice [78, 79]. Accordingly, the following hypothesis was formulated.

#### H5

**SI** has significant effects on health professionals’ attitudes toward mobile-based clinical guideline applications.

#### H6

**SI** has significant effects on health professionals’ BI to accept mobile-based clinical guideline applications.

### Facilitating conditions

Facilitating conditions (FC) is one of the constructor elements in the UTAUT model [56]. It is a belief that whether there is the availability of ICT, technical infrastructure, and trustworthy support in the organization for system users [56, 80]. FC provides system users with a sense of psychological control that in turn, influences their willingness to adopt a particular behavior. Hence, mobile-based clinical gaudiness-receiving users are required to have specific basic skills such as how to operate and use mobile phones, and how users react to the basic function of a mobile device (phone calls, sending and receiving text messages) [81, 82]. If system users do not have these required operational skills and basic mobile functions, they will not accept and adopt mobile-based clinical guidelines applications. So, the following hypothesis was developed.

#### H7

**FC** positively affects health professionals’ attitudes toward mobile-based clinical guideline applications.

#### H8

**FC** positively influences the health professionals’ acceptance of mobile-based clinical guideline applications.

### Computer literacy

Computer literacy (CL) is health professionals’ basic information communication technology skill and knowledge, the ability they have, and how system users are technically good at using mobile-based clinical guideline applications [60, 83]. An individual also can seek, evaluate, and communicate information using media across a range of digital platforms, and influence acceptance of mobile-based clinical guidelines applications [59, 84, 85].

#### H9

CL has a positive effect on health professionals’ attitudes toward mobile-based clinical guideline applications.

#### H10

CL has a positive effect on health professionals’ acceptance of mobile-based clinical guideline applications.

### Attitude

Attitude (ATT) is a psychological construct that shows how people think, feel, and tend to behave about an object or a phenomenon [86]. It is a predisposed state of mind regarding the importance of a new system in reducing workload, enhancing work performance, and accomplishing tasks efficiently and effectively [39, 87]. According to studies, attitude is appropriate in studying behavioural intention to accept and use new technologies, and it he one of the fundamental constructs for the successful implementation and adoption of a new technology [88–90]. Therefore, health professionals’ attitudes are crucial for the acceptance of mobile-based clinical guideline applications in the study setting.

#### H11

ATT directly affects the BI of health professionals’ acceptance of mobile-based clinical guideline applications.

#### H12

ATT mediates the relationship between PE and health professionals’ BI towards the acceptance of mobile-based clinical guideline applications.

#### H13

ATT mediates the relationship between EE and health professionals’ BI towards the acceptance of mobile-based clinical guideline applications.

#### H14

ATT mediates the relationship between SI and BI of health professionals to accept mobile-based clinical guideline applications.

#### H15

ATT mediates the relationship between FC and BI of health professionals to accept mobile-based clinical guideline applications.

#### H16

ATT mediates the relationship between CL and BI of health professionals to accept mobile-based clinical guideline applications.

### The effects of moderators (age, and sex)

Studies show in China that age has significant moderating effects on effort expectancy and behavioural intention to use health technology [62], home telehealth acceptance [69], and mobile health services adoption [63]. Other studies show that age has a moderating effect on performance and effort expectancy, social influence, and behavioural intention to use health information communication technology, smart equipment, and wearable devices [91, 92]. Similarly, sex has moderating effects on the modified UTAUT model’s construct elements [69, 93]. For instance, being female has a significant influence on the performance expectancy of behavioural intention to use wearable technology [93]. Therefore, the following hypotheses for moderators (age and sex) have been formulated.

#### H17

The effects of performance expectancy on health professionals’ intention to accept mobile-based clinical guideline applications has moderated by age.

#### H18

The effects of effort expectancy on health professional intention to accept mobile-based clinical guideline application has moderated by age.

#### H19

The effects of social influence on health professionals’ intention to accept mobile-based clinical guideline applications has moderated by age.

#### H20

The effects of facilitating conditions on health professional intention to accept mobile-based clinical guideline application moderated by age.

#### H21

The effects of computer literacy on health professionals’ intention to accept mobile-based clinical guideline applications has moderated by age.

#### H22

The effects of performance expectancy on health professional intention to accept mobile-based clinical guideline application has moderated by sex.

#### H23

The effects of effort expectancy on health professional intention to accept mobile-based clinical guideline application has moderated by sex.

#### H24

The effects of social influence on health professional intention to accept mobile-based clinical guideline application has moderated by sex.

#### H25

The effects of facilitating conditions on health professional intention to accept mobile-based clinical guideline application moderated by sex.

#### H26

The effects of computer literacy on health professionals’ intention to accept mobile-based clinical guideline applications have been moderated by sex.

### Methods

#### Study design

The institutional-based cross-sectional study design was employed among health professionals.

### Study setting and period

The study was done among health professionals working in the Ilu Aba Bora Zone of the Oromia regional state, from July 04 to August 19, 2022. Ilu Aba Bora Zone is found in Southwest Ethiopia. The zone is located 600 km away from Addis Ababa, the capital city of Ethiopia. The public health facilities provide different health services for more than a million of the population in southwest parts of Ethiopia.

### Study population and eligibility criteria

All healthcare professionals working in the public health facilities of the study area were the source population. All the healthcare professionals who were permanently employed were the study population. Healthcare professionals who were not present during the data collection period, who had a serious health problem, and on annual leave were excluded.

### Sample size determination and sampling procedures

The sample size was determined based on structural equation model parameter criteria which were considered the number of all variance of the independent variable, covariance of exogenous variables, direct and indirect regression coefficients between latent variables, and coefficient between latent and loading of the items. Accordingly, we estimated 33, 10, 16, and 14 free parameters in the hypothetical model respectively. Consequently, a total of 73 free parameters were determined in the model. In structural equation model analysis, a minimum of 10 sample sizes were required for the single free parameters [94, 95]. Hence, 730 sample sizes were required, and considering 10% of the non-response rate, a total of 803 sample sizes were estimated. A stratified simple random sampling method was used. Once the sample was stratified based on the types of facility, the sample was allocated in each stratum proportionally. Then, a simple random sampling technique was used to select the study subjects in each public health facility.

### Data collection and quality management

A pretested self-administered tool was used. The tool of the study was adapted in reviewing previously similar studies [39, 75, 96]. The tool had two parts: the first part contains sociodemographic characteristics of the study participants, and the second part contains key constructs of individuals’ behavioral intention of acceptance of technology in the UTAUT model [67]. The questionnaire was constructed to test the formulated hypothesis. As shown in SI 1, a total of 26 items of questions were used for the second part. Of these questions, 4 items were for “performance expectancy”, 4 items were for “effort expectancy”, 4 items were for “facilitating condition”, 4 items were for “computer literacy”, 4 items were for “attitude”, 3 items were for “social influence”, and 3 items were for “BI of acceptance”. All the items used to measure the key construct of BI were measured by using a Likert scale ranging from 1 (strongly disagree) to 5 (strongly agree). Two-day intensive training was delivered for the data collectors and supervisors. A pre-test was done outside of the study area (Buno Bedele Zone of Oromia region) with 10% of the total estimated sample units to check the readability and consistency of the tool. The data obtained from the pre-test was used to check the validity and reliability of the tool. Also, during the pertest health professionals’ experience of using mobile-based clinical guidelines was assessed. As a result, the study participants had no experience using mobile-based clinical guideline applications.

### Operationalization

#### Mobile-based clinical guideline applications

In this study, clinical guidelines are considered any clinical statements, guidelines, producers, and handbooks developed by governmental and nongovernmental agents and experts for assisting healthcare practitioners in making consistent and accurate evidence-based decisions. Therefore, properly handling these clinical guidelines using easily accessible mobile-based applications with a good format for accessibility and readability of clinical guidelines efficiently and effectively regardless of the health professional’s location [97, 98].

#### Health professionals

In this study, health professionals include certified health practitioners from known governmental and private institutions who are concerned with diagnosing, treating, and preventing human illness, injury, and other physical, social, and mental health issues by the needs of the populations they serve through the standard principles and procedures [99].

### Data processing and analysis

A statistical analysis technique based on the Structural Equation Model (SEM) was used to test and validate the formulated hypothesis. The data from the questionnaire were exported into SPSS software version 25. Amos version 26 software was used to analyze the data. Descriptive statistics of the study participants were calculated and presented with frequency and percentage Composite reliability was used to assess the internal reliability of each item of the constructs. The acceptable value of composite reliability (0.6) was considered for the internal reliability test [100, 101]. Convergent validity was assessed using an Average Variance Extracted (AVE) and factor loading. Hence, AVE for each associated construct should exceed 0.50, and the items loading above 0.6 [102, 103]. The discriminant validity was assessed using the Fornell Larcker criterion which is the square root of the AVE and cross-loading matrix. The square root of the AVE in the diagonal elements must be greater than the entire corresponding columns and rows to satisfy the discriminant validity [104]. To investigate the relationship between associated constructs, path coefficient (beta coefficients), 95% Confidence Interval, and p-value were used to check the hypothesis.

For moderator testing, the two model such as unconstrained, and constrained models were used. For both models, the moderator (age, sex) is assessed whether the moderator had an effect or significant difference for a given variable to influence the constructs and outcome variables. Accordingly, if a significant difference between the two models exists with p-value < 0.05. Then, the moderator confirmed that it had a significant effect on influencing other construct variables on the health professional’s intention to accept mobile-based clinical guidelines application.

## Results

### Socio-demographic characteristics of the study participants

A total of 769 health professionals participated in this study, and returned the questionnaire, with a 95.8% response rate. From the total of 769 respondents, around one-half (52%) of the respondents were males, and the majority (63%) of the respondents were degree and diploma holders. More than half of the respondents (55.7%) were less than 30 years of age, and the majority (62%) of the health professionals had up to ten years of work experience. Five out of eleven study participants (45.30%) had a monthly salary of < = 600 birrs (Table 1).

**Table 1:** Sociodemographic characteristics of study participants

Variable	Category	Frequency (*n*)	Percentage (%)
Sex	Male	400	52.00
	Female	369	48.00
Educational status	Degree and diploma	484	63.00
	Master	68	8.80
	Specialist and GP	217	28.20
Age (in years)	Less than 30 years	429	55.70
	30–40 years	242	31.50
	> 40 years	98	12.80
Experience	< 5 years	123	16.00
	5–10 years	477	62.00
	> 10 years	169	22.00
Month salary (Ethiopian birr)	<=6000	348	45.30
	6000–9000	189	24.60
	> 9000	232	30.10

### Descriptive results of the constructs of the modified UTAUT model

In this study, 46.9%, 53.3%, and 61.1% of health professionals strongly agreed and intended to learn, use, and plan to use their smartphones for mobile-based clinical guidelines applications, respectively. According to the participants’ computer literacy, 32.0%, 25.6%, and 27.0% of health professionals strongly disagree on properly searching information from the online database, correcting and fixing problems happening on their computers and smartphones, and downloading and installing applications, respectively. However, 31.9% of the participants strongly disagree that they would lack the skills to practice and use the basic functions of computers and smartphones they have. According to participants’ attitudes, 46.2%, 48.5%, 45.5%, and 49.5% of participants agreed that mobile-based clinical guideline applications would be important to access the right information, useful for quality, and consistency of patient care, and they would not hesitate and fear to use the application, respectively. According to facilitating conditions, 33.1% and 36.5% of participants strongly disagreed that they would lack adequate skills and knowledge to use the application and that the application would not be compatible with their smartphone, respectively. Also, 56.4% and 43.1% of participants strongly disagreed with the resources they have, and the supportiveness of the organization to use the application, respectively.

According to social influence, 39.8%, 42.8%, and 37.3% of the participants strongly agreed that people’s influence, motivation, and options would be important to use mobile-based clinical guideline applications, respectively. According to effort expectancy, 49%, 38.8%, 54.7%, and 43.3% of the study participants strongly agree that mobile-based clinical guideline applications would be easy to use, not difficult, clear, and understandable, and would allow the practitioners to become skilful, respectively. According to performance expectancy, 30.9%, 42,7%, 43.6%, and 31.7% of the participants agreed that mobile-based clinical guideline applications would be useful to use, enable them to share information and update themselves, supportive for accurate and consistent patient care, and it wound to ensure the quality of patient care with low waiting time, respectively (**SI 2**).

### Measurement model

The convergent validity of the structural model assessment is presented in Table 2. Based on the results, the internal consistency of each item of the latent variable was assessed by composite reliability. Composite reliability is acceptable and considered good if it ranges between 0.60 and 0.90 [104, 105]. As a result, values of composite reliability of the latent variables ranged from a minimum of 0.750 to a maximum of 0.890, and this indicated that the respondents’ answers for each item of the latent variable were consistent and had strong internal reliability. Factor loading values of each latent variable range from a minimum of 0.63 to a maximum of 0.96. This showed that each latent variable was greater than a minimum acceptable value (0.6). The degree of variation of each latent variable was measured by the average variance extracted (AVE) value. Consequently, the analysis values of AVE ranged from a minimum of 0.582 to a maximum of 0.778. Hence, each latent variable has an estimated strong power variation between them. Consequently, the conditions for convergent validity were satisfied in this study. Furthermore, the factor loading of each item was significant on its respective construct (p-value < 0.001).

**Table 2:** Constructs’ convergent validity for healthcare professionals’ acceptance of mobile-based clinical guidelines in a resource-limited setting, northwest Ethiopia 2023

Latent variables	Indicator/items	Factor loading	Composite reliability	AVE	Convergent validity
Performance Expectancy (PE)	PE1	0.63	0.792	0.683	Established
	PE2	0.68			
	PE3	0.82			
	PE4	0.84			
Effort Expectancy (EE)	EE1	0.85	0.769	0.591	Established
	EE2	0.72			
	EE3	0.75			
	EE4	0.68			
Social Influence (SI)	SI1	0.73	0.817	0.710	
	SI2	0.80			
	SI3	0.79			
Facilitating Condition (FC)	FC1	0.91	0.750	0.582	Established
	FC2	0.65			
	FC3	0.69			
	FC4	0.86			
Attitude (ATT)	ATT1	0.71	0.852	0.720	Established
	ATT2	0.76			
	ATT3	0.79			
	ATT4	0.80			
Computer literacy	CL1	0.68	0.759	0.580	Established
	CL2	0.65			
	CL3	0.69			
	CL4	0.63			
Behavioral Intention	BI1	0.96	0.890	0.778	Established
	BI2	0.76			
	BI3	0.89			

AVE: Average variance extracted

The results of discriminant validity or divergent validity between different constructs are presented in Table 3. The elements in the matrix diagonals represent the square roots of the AVEs and are greater than the values in their corresponding row and column. As a result, all constructs in this study supported the discriminant validity of the data (Table 3).

**Table 3:** Divergent validity

Constructs	PE	EE	SI	FC	BI	CL	ATT	Divergent validity
PE	**0.826**							Established
EE	0.306	**0.769**						Established
SI	−0.021	0.259	**0.843**					Established
FC	0.278	0.195	−0.008	**0.763**				Established
BI	0.359	0.459	0.207	0.277	**0.882**			Established
CL	0.245	0.103	−0.027	0.201	0.170	**0.762**		Established
ATT	0.166	0.267	0.200	0.153	0.284	0.227	**0.849**	Established

PE: Performance expectancy, EE: Effort expectancy, SI: Social influence, FC: Facilitating conditions, ATT: Attitudes, CL: Computer literacy, BI: Behavioural intention

### Model goodness of fit

The model goodness of fit the data was checked using Chi-squire (P-value < 0.05), goodness of fit indices (GFI > 0.9), adjusted goodness of fit indices (AGFI > 0.8), normal fit indices (NFI > 0.95), Tucker–Lewis index (TLI > 0.9), comparative fit indices (CFI > 0.95), root mean square of standardized residual (RMSSR < 0.08), and (RMR < 0.08) model fit indices assessment criteria [86, 106]. To say that the model goodness of fit is achieved, the value of Chi-squire, GFI, AGFI, TLI, RMSEA, and RMR should fulfil the cut-off point. As a result, all the required criteria were achieved and the data fitted the goodness of the model (Table 4).

**Table 4:** Model goodness of fit assessment

Model fit indices	Cut-off point	Result obtained	Conclusion
**Chi squire**	< 3	2.72	Supported
Goodness-of-fit-index **(GFI)**	> 0.9	0.95	Supported
Adjusted goodness-of-fit-index (AGFI)	> 0.8	0.87	Supported
Comparative fit index **(CFI)**	> 0.95	0.97	Supported
Tucker–Lewis index **(TLI)**	> 0.9	0.94	Supported
Root mean square error of approximation **(RMSEA)**	< 0.08	0.06	Supported
Root mean squared residual **(RMR)**	< 0.08	0.05	Supported

#### The structural model analysis

**Fig. 2:**
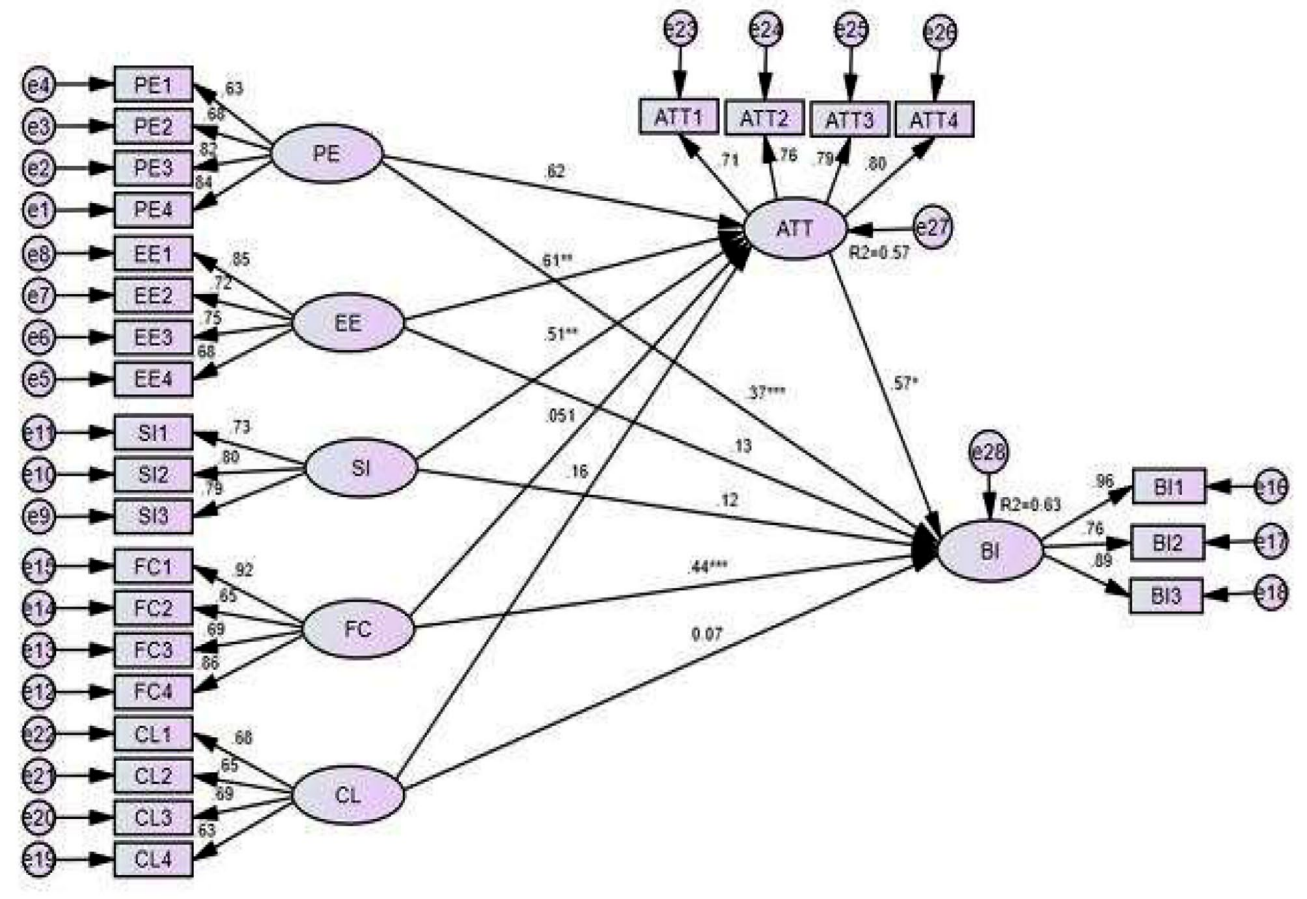
Results of the structurally modified UTAUT model. *, **, and *** indicates significant at P-value < 0.05, 0.01, and 0.001, respectively. **PE**: Performance expectancy, **EE**: Effort expectancy, **SI**: Social influence, **FC**: Facilitating conditions, **ATT**: Attitudes, **CL**: Computer literacy, **BI**: Behavioral intention

As shown in Table 5, the analysis report of the structural model showed that performance expectancy, facilitating condition, and computer literacy did not have any positive effects on health professionals’ attitudes toward mobile-based clinical guideline applications. Plus, facilitating conditions and computer literacy had not had any positive effects on health professionals’ BI toward acceptance of mobile-based clinical guideline applications. Effort expectancy and social influence had a significant effect on health professionals’ attitude toward mobile-based clinical guideline application with path coefficient (B-coefficient) of (β = 0.61, P-value < 0.01), and (β = 0.510, P-value < 0.01) respectively. Performance expectancy, facilitating condition, and attitude had a significant effect on health professionals’ BI of mobile-based clinical guideline application acceptance with path coefficient (B-coefficient) of (β = 0.37, P-value < 0.001), (β = 0.44, P-value < 0.001) and (β = 0.57, P-value < 0.05) respectively. All the latent variables such as performance expectancy, effort expectancy, social influence, facilitating condition, and computer literacy accounted for 57% of health professionals’ attitudes toward mobile-based clinical guideline application. All the latent variables such as performance expectancy, effort expectancy, social influence, facilitating condition, and computer literacy including health professionals’ attitude accounted for 63% of health professionals’ BI of mobile-based clinical guideline application acceptance (Fig. 2).

**Table 5:** Result of structural model analysis

Path	Β	SE	Critical ratio	*P*-value	Decision
PE-> ATT	0.620	0.036	1.207	0.227	Not supported
PE-> BI	0.366	0.018	4.490	0.001***	Supported
EE-> ATT	0.614	0.012	3.048	0.002**	Supported
EE-> BI	0.130	0.163	1.490	0.208	Not supported
SI-> ATT	0.510	0.037	2.950	0.003**	Supported
SI-> BI	0.124	0.049	1.529	0.111	Not supported
FC-> ATT	0.051	0.054	1.509	0.131	Not supported
FC-> BI	0.443	0.024	3.214	0.001***	Supported
CL-> ATT	0.159	0.059	1.234	0.201	Not supported
CL-> BI	0.065	0.164	1.023	0.306	Not supported
ATT-> BI	0.574	0.019	2.188	0.029*	Supported

*Significant at *P* < 0.05, **Significant at *P* < 0.01, ***Significant at *P* < 0.001; PE: Performance expectancy, EE: Effort expectancy, SI: Social influence, FC: Facilitating conditions, ATT: Attitudes, CL: Computer literacy, BI: Behavioral intention

### Mediation analysis

In the mediation analysis shown in Table 6, the relationship between effort expectancy, and health professionals’ acceptance of mobile-based clinical guideline application had a significant partial mediation with attitude. In addition, the relationship between social influence, and health professionals’ acceptance of mobile-based clinical guideline applications had a significant partial mediation with attitude. Accordingly, effort expectancy and social influence had an indirect effect relationship with health professionals’ BI towards mobile-based clinical guidelines application acceptance with standardized estimation coefficient (**β = 0.22, P-value = 0.027**), and (**β = 0.19, P-value = 0.031**), respectively.

**Table 6:** Mediation analysis result

Path	Hypothesis	Effect	Estimate	*P*-value	Results	Decision
PE-> ATT-> BI	H12	Total	0.234	0.002	Direct relationship	Not supported
		Indirect	0.008	0.206		
		Direct	0.226	0.002		
EE-> ATT-> BI	H13	Total	0.355	0.000	Partial mediation	Supported
		Indirect	0.25	0.027		
		Direct	0.333	0.000		
SI > ATT-> BI	H14	Total	0.150	0.020	Partial mediation	Supported
		Indirect	0.190	0.031		
		Direct	0.130	0.037		
FC-> ATT-> BI	H15	Total	0.166	0.003	Direct relationship	Not supported
		Indirect	0.010	0.218.		
		Direct	0.157	0.004		
CL-> ATT-> BI	H16	Total	0.078	0.185	Indirect relationship	Not supported
		Indirect	0.023	0.036		
		Direct	0.055	0.371		

### Moderating effects of sex and age of health professionals on intention to accept mobile-based clinical guideline application

The effects of sex, and age on the relationship between performance expectancy, effort expectancy, social influence, facilitating conditions, and computer literacy with health professionals’ intention to accept mobile-based clinical guideline applications was investigated. The moderators were estimated both in constrained and unconstrained models.

Accordingly, performance expectancy, facilitating conditions, and social influence on health professionals’ intention to accept mobile-based clinical guideline applications had not significantly moderated by the sex of health professionals. However, computer literacy and effort expectancy on health professionals’ intention to accept mobile-based clinical guideline applications was significantly moderated by sex. Being male had a significant effect on the effort expectancy of health professionals’ intention to accept mobile-based clinical guideline applications with a path coefficient of 0.712 and a p-value of 0.018. Being female also had a significant effect on the computer literacy of health professionals’ intention to accept mobile-based clinical guideline applications with a path coefficient of 0.316 and a p-value of 0.001 (Table 7). Therefore, H23 and H26 were supported in this study.

**Table 7:** The moderating effect of the sex of healthcare professionals on the intention to accept mobile-based clinical guideline application

Path	Moderator	Path coefficient	P-value	Model test (constructed & unconstructed difference)		Remarks
				Δ X^2^	P-value	
PE-> BI	Male	0.310	0.257	0.413	0.814	Not supported
	Female	0.342	0.313			
EE-> BI	Male	0.712	0.018*	8.431	0.005**	Supported
	Female	0.351	0.144			
SI-> BI	Male	0.021	0.741	3.101	0.412	Not supported
	Female	0.400	0.082			
FC-> BI	Male	0.56	0.612	0.812	0.671	Not supported
	Female	0.84	0.411			
CL-> BI	Male	0.915	0.101	6.513	0.002**	Supported
	Female	0.316	0.011*			

For measuring the effects of age on the constructs, average age [36] was used as a cut-off point to dichotomize age as young (< 36 years) and old (≥ 36 years). Therefore, age had a significant effect on the computer literacy of health professionals’ intention to accept mobile-based clinical guideline applications, where young health professionals positively influenced health professionals’ acceptance of mobile-based clinical guideline applications with a path coefficient of 0.718, and a p-value of 0.031(Table 8). Therefore, H21 was supported.

**Table 8:** The moderating effect of the age of healthcare professionals on the intention to accept mobile-based clinical guideline application

Path	Moderator	Path coefficient	P-value	Model test (constructed & unconstructed difference)		Remarks
				Δ X^2^	P-value	
PE-> BI	Old	0.421	0.504	0.465	0.700	Not supported
	Young	0.648	0.616			
EE-> BI	Old	0.658	0.091	6.815	0.217	Not supported
	Young	0.531	0.416			
SI-> BI	Old	0.020	0.602	4.133	0.182	Not supported
	young	0.714	0.052			
FC-> BI	Old	0.675	0.615	0.712	0.719	Not supported
	Young	0.496	0.342			
CL-> BI	Old	0.548	0.228	5.813	0.0141*	Supported
	Young	0.718	0.031*			

## Discussion

This study was conducted to determine the effects of constructs of the UTAUT model on health professionals’ acceptance of mobile-based clinical guideline applications before the actual use of the applications. In this study total of 803 health professionals participated. Therefore, the study was different from other similar studies in terms of the representative sample size used, which is important to save resources to make decisions based on this study. In addition, the study verified that the constructs (PE, EE, SI, FC, CL, and ATT) of the UTAUT model would explain individuals’ attitudes towards mobile-based clinical guidelines application and health professionals’ acceptance before the actual use of the application. In this study, convergent and divergent validity were assessed, and the model goodness of fit was also tested. As a result, all the mentioned criteria of the structural equation model were achieved.

A hypothesis for all the constructs was formulated, and their effects on the health professionals’ acceptance of mobile-based clinical guidelines applications were checked. As a result, performance expectancy, facilitating conditions, and computer literacy had no positive effects on health professionals’ attitudes toward mobile-based clinical guidelines application (**H1**, **H7**, and **H9**). Additionally, facilitating conditions and computer literacy had no positive effects on health professionals’ acceptance of mobile-based clinical guidelines (**H8** and **H10**). Performance expectancy and effort expectancy had a significant effect on health professionals’ behavioral intentions, and attitudes toward mobile-based clinical guideline applications, respectively (H2 and H3). Plus, facilitating conditions and social influence had a significant effect on health professionals’ Behavioral intentions, and attitudes towards mobile-based clinical guideline application acceptance, respectively (**H8** and **H5**). According to hypothesis **H11**, health professionals’ attitudes had a direct effect on their Behavioral intentions toward the mobile-based clinical guidelines application. In the mediation analysis result, effort expectancy and social influence had a significant indirect and standardized partial relationship with health professionals’ acceptance of mobile-based clinical guidelines applications.

Effort expectancy had a significant effect on health professionals’ attitudes towards mobile-based clinical guideline applications, and its relationship with health professionals’ acceptance of mobile-based clinical guideline applications was mediated by the health professionals’ attitudes. This finding was supported by similar studies conducted in different geographical areas [107, 108]. Other studies also proved that effort expectancy had a significant influence on the adoption of healthcare information technology, and MHealth applications [71, 108, 109]. The finding opposes a study report that states mobile applications are difficult to use, the benefits of using mobile applications are offset by the effort to use the mobile application, as well as the more complex an innovation is, the lower its rate of acceptance, and adoption of the mobile-based clinical guideline application again [110, 111]. However, effort expectancy has a positive influence on individuals’ acceptance of new technology (mobile-based clinical guideline application), and its indirect effect on attitude [112]. This might be due to health professionals’ attitudes, the belief that using the new application is easy, and the intention to use mobile-based clinical guideline applications positively influenced by the effort made to use mobile applications [39]. Plus, effort expectancy is associated with diagnosis and medication error reduction [113], applications’ flexibility, friendliness, familiarity, and its easiness of individuals to use. Additionally, mobile phones are now routinely used in education, entertainment, communication, and healthcare facilities [67]. So, it might not need too much effort, and users might not face technical problems.

The social influence had a significant effect on health professionals’ attitudes toward mobile-based clinical guideline applications, and its relationship with health professionals’ acceptance of mobile-based clinical guideline applications was mediated by the health professionals’ attitudes. This was congruent with other similar studies [60, 75, 86, 114]. It was concluded that the viewpoints and opinions of others regarding the use of information technology in education and learning were affected by health professionals’ behavioral intentions for the frequent and daily use of technology [115]. This is associated with expert clinical guideline development skills for disease management and might influence individual health professionals’ acceptance of mobile-based clinical guideline applications [116].

Performance expectancy had a significant effect on health professionals’ acceptance of mobile-based clinical guideline applications. This could be because mobile-based clinical guidelines applications could be useful for assisting health professionals in monitoring the disease progression of the patient and managing disease [117]. Additionally, mobile clinical guidelines applications could also provide health professionals with real-time information on the patient’s specific health condition [118, 119]. So, mobile-based clinical guidelines could be effective for better healthcare outcomes. Performance expectancy enhances the productivity of health professionals and is efficient for the time spent in operation, patient management, and the care provider’s intention and attitude toward mobile-based clinical guideline application acceptance [39]. This study’s findings were similar to those of previous studies [72, 120, 121].

The facilitating conditions had a significant effect on health professionals’ BI of mobile-based clinical guideline application acceptance. This finding was consistent with similar studies conducted in Ethiopia [60, 86], Nigeria [122], South Africa [123], and Malaysia [124]. Facilitating conditions such as organizational setting, preliminary skill, and knowledge they had on a mobile device, resources, and availability of training for information sharing [122], and system quality might have an important role in predicting users’ actual acceptance of mobile-based clinical guideline applications [86]. All these facilitating conditions might be user-friendly, comprehensive, and easily available for mobile-based clinical guidelines application acceptance by individuals.

Attitude had a significant effect on health professionals’ acceptance of mobile-based clinical guideline applications. This finding was consistent with previous studies [39, 86]. This might be because health professionals’ attitudes toward using mobile-based systems have improved over time, and individuals’ sociodemographic characteristics and educational level affect their attitudes which further affect their behavioral intention of technology acceptance [125, 126].

### Conclusions and recommendations

This study reported that the unified theory of acceptance and use of technology (UTAUT) model proved a suitable model to assess health professionals’ attitudes and behavioral intentions towards the acceptance of mobile-based clinical guidelines applications. Social influence, effort expectancy, and facilitating conditions were significant constructs for health professionals’ acceptance of mobile-based clinical guideline applications. Health professionals’ attitude toward mobile-based clinical guideline application was another strong construct in the UTAUT model for the acceptance of mobile-based clinical guidelines. Plus, effort expectancy and social influence had a positive effect on health professionals’ attitudes toward mobile-based clinical guideline applications. The development of user-friendly mobile-based clinical guideline applications, based on user’s requirements and in line with national standards of clinical guidelines, would be encouraged for consistent and accurate health professionals’ decision-making processes. So, stakeholders and policymakers are advised to build the capacity and technical skills of health professionals to enhance their overall computer literacy. Moreover, resources and organizational support of health professionals would be critical for the acceptance of mobile-based clinical guideline applications.

### Implications of the study and future research directions

#### Theoretical implications

This study contributes to the growing body of literature on the application of mobile devices for healthcare practice and education promotion. The applied extended UTAUT model was proven to be suitable for predicting mobile-based clinical guideline acceptance. This study assessed the acceptance of mobile-based clinical guideline applications among health professionals’ perspectives, which aided in the development and enhancement of locally relevant clinical practice guidelines. This study may alleviate any concerns of readers about the UTAUT model, and mobile-based clinical guidelines, and it serves as a baseline for researchers since there is insufficient evidence on a similar topic.

#### Practical implications

This study provides valuable implications for fostering the future implementation of mobile-based clinical guidelines. Based on the significant predictors, the current study may be important to offer tailored programs to increase users’ digital knowledge and to ensure that using mobile-based clinical guidelines applications is easy and simple. Performance expectancy is a significant predictor of the acceptance of mobile-based clinical guidelines. This indicates that it is vital to demonstrate the advantages of mobile-based clinical guidelines to healthcare professionals.

#### Implications for future research direction

Future research should therefore concentrate on approaches to simplifying the acceptance level of mobile-based clinical guidelines, and removing technical barriers. Future research should focus on exploring further suitable and specific predictors to enhance the viability of the UTAUT model in a health-related context. The proposed predictors could also easily be applied in studies on the actual use of locally available mobile-based systems in healthcare practice that enable researchers to examine their ultimate predictive power. Researchers are also encouraged to conduct similar studies on governmental and non-governmental health institutions. Decision makers, care healthcare providers, and system developers could use this study’s findings to increase the adoption of mobile-based clinical guidelines in the future.

### Strengths and limitations of the study

This study will provide input for future research and mobile-based clinical guidelines application implementation and adoption in low-income settings. Additionally, this study proved that constructs in the UTAUT model affect health professionals’ intention to accept new technology. Since the study is cross-sectional, there might be a temporal relationship between the effects of constructs and individuals’ behavioral intentions to accept mobile-based clinical guidelines applications. This study did not attempt to control the impact of confounding variables on the health professionals’ intention to accept mobile-based clinical guideline applications.

### Electronic supplementary material

Below is the link to the electronic supplementary material.

Supplementary Material 1

Supplementary Material 2

## Data Availability

All the data generated, and analyzed during the study are included in this article.

## Abbreviations

BI: Behavioural intention
EE: Effort expectancy
CL: Computer literacy
ICT: Information communication technology
TAM: Technology acceptance model
UTAUT: Unified theory of acceptance and technology use
PE: Performance expectancy
SI: Social influence
