# Supplementary material for: Health professionals’ acceptance of mobile-based clinical guideline application in a resource-limited setting: using a modified UTAUT model
Source: BMC Med Educ. 2024 Jun 25;24:689. doi: 10.1186/s12909-024-05680-z (PMC11202359; doi:10.1186/s12909-024-05680-z)
Supplement: Supplementary file 1 — Supplementary Material 1 [file 12909_2024_5680_MOESM1_ESM.docx]

**Supplementary file 1:** Questionnaire for constructs of UTAUT model and sociodemographic characteristics of the study participant.

| **Part one:** Sociodemographic characteristics of the study participants | | | | | | | |
| --- | --- | --- | --- | --- | --- | --- | --- |
| 1. 1. | Sex | 1. Male 2. Female | | | | | |
| 2. | Educational status | 1. Diploma 2. Degree 3. Masters 4. GP 5. Specialist | | | | | |
| 3. | Age by years | _________________ | | | | | |
| 4. | Work experience | ____________________________ | | | | | |
| 5. | Month salary (Ethiopian birr) | _____________________ | | | | | |
| **Part two:** Questions for the constructs of the UTAUT model for health professionals’ acceptance of Mobile-Based Clinical Guidelines Application (MBCGA). | | | | | | | |
| **Effort expectancy** | | | | | | | |
| **Items** | **Questions** | | **Strongly disagree** | **Disagree** | **Neutral** | **Agree** | **Strongly agree** |
| EE1 | I would found that use of MBCGA would be easy to use. | |  |  |  |  |  |
| EE2 | Learning to operate MBCGA would not be difficult. | |  |  |  |  |  |
| EE3 | Working with MBCGA would be easy, clear, and understandable. | |  |  |  |  |  |
| EE4 | It would be easy to become skilful through using MBCGA at work place? | |  |  |  |  |  |
| Performance expectancy | | | | | | | |
| PE1 | I would found that using MBCGA is useful for my job. | |  |  |  |  |  |
| PE2 | Using the MBCGA enables me to read, share information and update myself. | |  |  |  |  |  |
| PE3 | Using the MBCGA would be supportive for accurate and consistent patient care. | |  |  |  |  |  |
| PE4 | MBCGA would help to ensure quality of patient care with low waiting time. | |  |  |  |  |  |
| Facilitating condition | | | | | | | |
| FC1 | I have necessary resource to use the MBCGA. | |  |  |  |  |  |
| FC2 | I have not adequate skill and knowledge to use MBCGA. | |  |  |  |  |  |
| FC3 | MBCGA would not be compatible with my smart phone I have, and there is supportive technical staff that would fix the problem happen. | |  |  |  |  |  |
| FC4 | The organization manger is generally supportive to use the application (MBCGA). | |  |  |  |  |  |
| Social influence | | | | | | | |
| SI1 | People who influence my behaviour think that I should use the application (MBCGA). | |  |  |  |  |  |
| SI2 | Peoples’ motivation would be important for me to use the application (MBCGA). | |  |  |  |  |  |
| SI3 | People whose opinions could be critical to use the application (MBCGA). | |  |  |  |  |  |
| Attitude towards the application (MBCGA). | | | | | | | |
| ATT1 | I think that MBCGA would be important to access the right information when I need it. | |  |  |  |  |  |
| ATT2 | I believe that using mobile application (MBCGA) would be useful for the quality of patient care. | |  |  |  |  |  |
| ATT3 | I think that using mobile application (MBCGA) would improve the consistence of patient care. | |  |  |  |  |  |
| ATT4 | Do you hesitate and fear of using mobile application (MBCGA)? | |  |  |  |  |  |
| Computer literacy | | | | | | | |
| CL1 | Do you lack skills to practice and use basic function of computer and smart phone? | |  |  |  |  |  |
| CL2 | I can properly search information from computer and online database. | |  |  |  |  |  |
| CL3 | I can correct and fix problems happen in my computer and smartphone. | |  |  |  |  |  |
| CL4 | I can properly download and install application software on my personal computer and smart phone. | |  |  |  |  |  |
| Behavioral intention towards acceptance of the application (MBCGA). | | | | | | | |
| BI1 | I am highly intendent to learn the mobile application (MBCGA) | |  |  |  |  |  |
| BI2 | I am highly intended to use the mobile application (MBCGA) | |  |  |  |  |  |
| BI3 | I planned to use the smart phone I have for healthcare practice as a supporting tool. | |  |  |  |  |  |

MBCGA: Mobile-based clinical guidelines applications.
