# Supplementary material for: Health professionals’ acceptance of mobile-based clinical guideline application in a resource-limited setting: using a modified UTAUT model
Source: BMC Med Educ. 2024 Jun 25;24:689. doi: 10.1186/s12909-024-05680-z (PMC11202359; doi:10.1186/s12909-024-05680-z)
Supplement: Supplementary file 2 — Supplementary Material 2 [file 12909_2024_5680_MOESM2_ESM.docx]

| **Supplementary file 2:** Health professionals' score for each construct of the UTAUT model | | | | | | |
| --- | --- | --- | --- | --- | --- | --- |
| **Latent variables** | **Indicator/ Items** | **Strongly disagreed** | **Disagree** | **Neutral** | **Agree** | **Strongly agree** |
| **Performance**  **Expectancy (PE)** | PE1 | 139 (18.1) | 164 (21.3) | 122 (15.9) | 238 (30.9) | 106 (13.8) |
|  | PE2 | 90 (11.7) | 119 (15.6) | 104 (13.5) | 329 (42.7) | 127 (16.5) |
|  | PE3 | 105 (13.7) | 35 (4.6) | 69 (9.0) | 335 (43.6) | 225 (29.3) |
|  | PE4 | 82 (10.7) | 100 (13.0) | 165 (21.5) | 244 (31.7) | 178 (23.1) |
| **Effort Expectancy (EE)** | EE1 | 73 (9.5) | 40 (5.2) | 114 (14.8) | 165 (21.5) | 377 (49.0) |
|  | EE2 | 43 (5.6) | 76 (9.9) | 168 (21.8) | 184 (23.9) | 298 (38.8) |
|  | EE3 | 97 (12.6) | 39 (5.1) | 103 (13.4) | 109 (14.2) | 421 (54.7) |
|  | EE4 | 31 (4.0) | 77 (10.0) | 161(20.9) | 168 (21.8) | 332 (43.3) |
| **Social Influence (SI)** | SI1 | 56 (7.3) | 81 (10.5) | 36 (4.7) | 290 (37.7) | 306 (39.8) |
|  | SI2 | 56 (7.3) | 109 (14.2) | 39 (4.7) | 239 (31.1) | 329 (42.8) |
|  | SI3 | 76 (9.9) | 123 (16.0) | 49 (6.4) | 234 (30.4) | 287 (37.3) |
| **Facilitating Condition (FC)** | FC1 | 434 (56.4) | 173 (22.5) | 41 (5.3) | 63 (8.2) | 58 (7.6) |
|  | FC2 | 254 (33.1) | 108 (14.0) | 101 (13.1) | 104 (13.5) | 202 (26.3) |
|  | FC3 | 281 (36.5) | 96 (12.5) | 84 (10.9) | 138 (17.9) | 170 (22.1) |
|  | FC4 | 331 (43.1) | 77 (10.0) | 161(20.9) | 68 (8.8) | 132 (17.2) |
| **Attitude (ATT)** | ATT1 | 34 (4.4) | 355 (46.2) | 83 (10.8) | 283 (36.8) | 14 (1.8) |
|  | ATT2 | 10 (1.3) | 373 (48.5) | 109 (14.2) | 34 (4.4) | 243 (31.6) |
|  | ATT3 | 38 (4.9) | 350 (45.5) | 170 (22.1) | 13 (1.7) | 198 (25.7) |
|  | ATT4 | 38 (4.9) | 381 (49.5) | 83 (10.8) | 44 (5.7) | 223 (29.0) |
| **Computer Literacy** | CL1 | 245 (31.9) | 116 (15.1) | 124 (16.1) | 118 (15.3) | 166 (21.6) |
|  | CL2 | 246 (32.0) | 142 (18.5) | 131 (17.0) | 151 (19.6) | 99 (12.9) |
|  | CL3 | 197 (25.6) | 156 (20.3) | 135 (17.6) | 167 (21.7) | 114 (14.8) |
|  | CL4 | 208 (27.0) | 136 (17.7) | 155 (20.2) | 147 (19.1) | 123 (16.0) |
| **Behavioural Intention** | BI1 | 26 (3.4) | 130 (16.9) | 46 (6.0) | 361(46.9) | 206 (26.8) |
|  | BI2 | 42 (5.5) | 162 (21.1) | 90 (11.7) | 410 (53.3) | 65 (8.5) |
|  | BI3 | 24 (3.1) | 126 (16.4) | 56 (7.3) | 470 (61.1) | 93 (12.1) |

*Note that what each indicator or item means in this table is clearly available in the questionnaire that is cited as a supplementary file in main manuscript at page 11.
